# Isolated and Localized Immunoglobulin 4–Related Cholecystitis Mimicking Gallbladder Carcinoma

**DOI:** 10.14309/crj.0000000000001576

**Published:** 2024-12-27

**Authors:** Kenji Yorita, Sunao Uemura, Michiyo Okazaki

**Affiliations:** 1 Department of Diagnostic Pathology, Japanese Red Cross Kochi Hospital, Kochi, Japan; 2 Department of Surgery, Japanese Red Cross Kochi Hospital, Kochi, Japan; 3 Department of Internal Medicine, Japanese Red Cross Kochi Hospital, Kochi, Japan

## CASE REPORT

**Figure 1.**
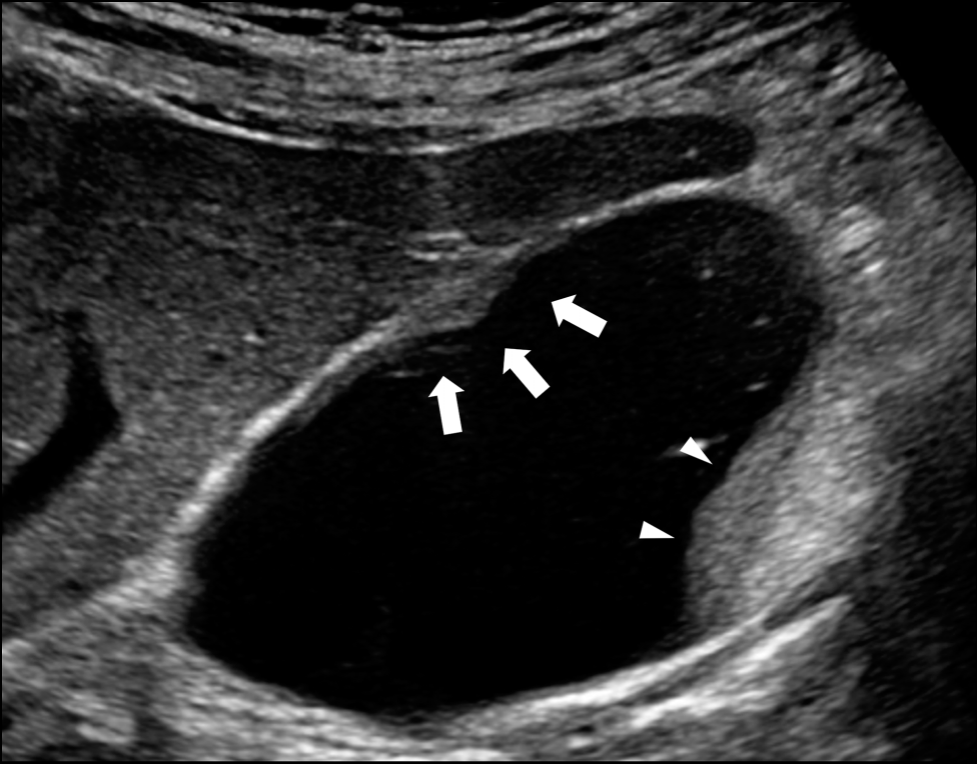
Abdominal ultrasonography. The gallbladder shows localized mural thickening (white arrows) with loss of a hyperechoic area at the liver-bed portion. Bile sludge (arrowheads) is observed.

**Figure 2.**
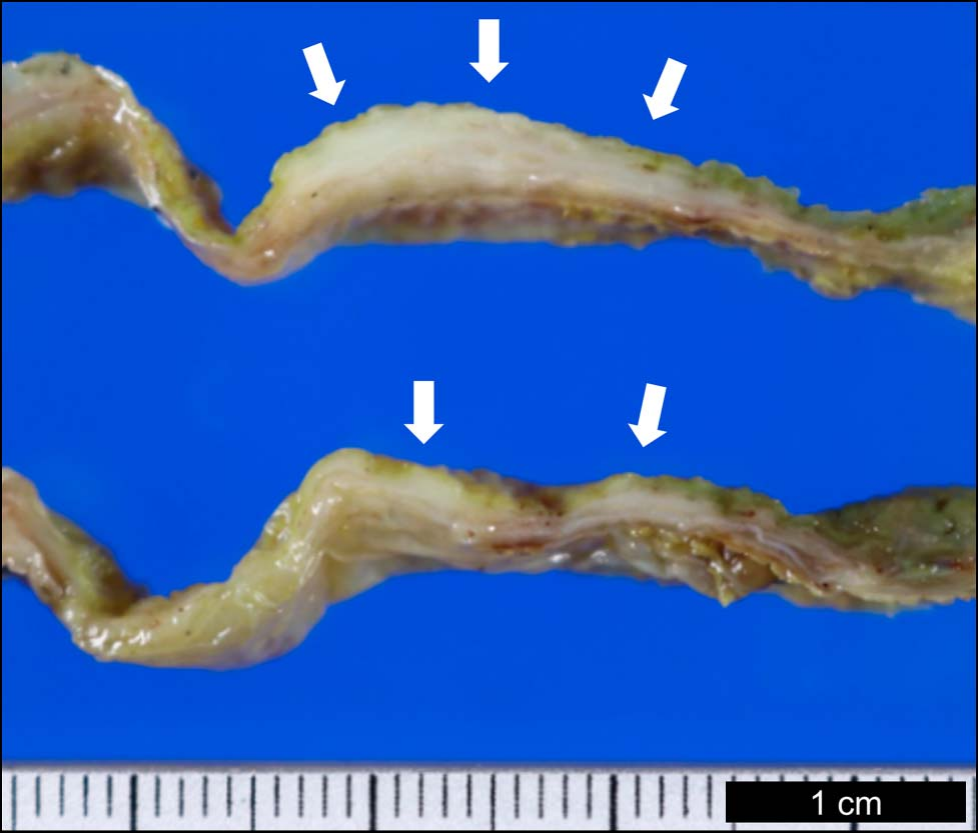
Gross findings of the gallbladder lesion. The gallbladder lesion shows a whitish, localized, and thickened wall (white arrows).

**Figure 3.**
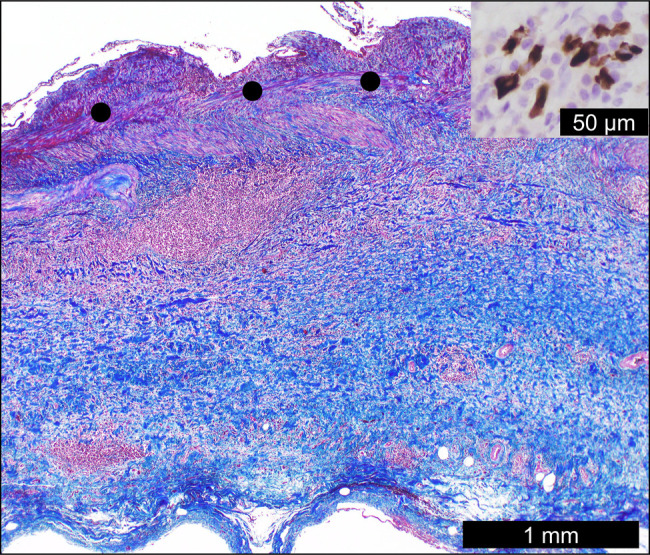
Microscopic findings of the gallbladder lesion. Masson trichrome staining confirms that the gallbladder lesion represents a mural fibrosis, particularly in the subserosa, and lymphoplasmacytic infiltrates containing immunoglobulin G4–positive plasma cells (inset). Black dots represent the muscularis propria.

Abdominal ultrasonography in a 78-year-old man with a history of abdominal pain confirmed localized mural thickening (Figure 1, white arrows), loss of hyperechoic area at the liver-bed portion, and the presence of bile sludge (Figure 1, arrowheads). Gallbladder cancer with subserosal invasion could not be ruled out because of the loss of the hyperechoic area,^1^ leading to cholecystectomy. Macroscopically, the gallbladder lesion showed a whitish, localized, and thickened wall (Figure 2, white arrows). Microscopically, mural fibrosis, particularly in the subserosa, was confirmed by Masson trichrome staining (Figure 3, blue area represents fibrosis; black dots represent the muscularis propria). Furthermore, obliterative phlebitis and numerous lymphoplasmacytic infiltrates containing immunoglobulin G4 (IgG4)–positive plasma cells were observed (Figure 3, inset), with a density of 62 cells per high-power field. The IgG4:IgG ratio was 47%. Postoperative radiological examination revealed no evidence of IgG4-related disease in any organs. The serum IgG4 levels (55 mg/dL; normal: 11–121 mg/dL) were normal. Isolated and localized IgG4-related cholecystitis mimicking malignancy, as seen in our case, has rarely been reported and may show normal serum IgG4 levels, complicating preoperative diagnosis.^2,3^ Autoimmune pancreatitis was not observed.^4^ Clinicians should consider IgG4-related cholecystitis in diagnostic assessments and monitor for potential IgG4-related disease in other organs.^5^

## DISCLOSURES

Author contributions: K. Yorita: Acquisition of data, analysis and interpretation of data, pathological diagnosis, and drafting of the manuscript; S. Uemura: acquisition of data, surgical treatment of the gallbladder lesion, and revising the draft critically for important intellectual content; M. Okazaki: detection of the gallbladder lesion, postoperative clinical examination, acquisition of written informed consent from the patient, and revising the draft critically for important intellectual content. K. Yorita is the article guarantor.

Financial disclosure: None to report.

Informed consent was obtained for this case report.

## References

[1] Fujita N Noda Y Kobayashi G Kimura K Yago A Mochizuki F. Analysis of the layer structure of the gallbladder wall delineated by endoscopic ultrasound using the pinning method. Dig Endosc. 2007;7(4):353–6.

[2] Feely MM Gonzalo DH Corbera M Hughes SJ Trevino JG. IgG4-related cholecystitis presenting as biliary malignancy: Report of three cases. J Gastrointest Surg. 2014;18(9):1710–5. 24944152 10.1007/s11605-014-2568-3

[3] Harada Y Mihara K Amemiya R Isolated IgG4-related cholecystitis with localized gallbladder wall thickening mimicking gallbladder cancer: A case report and literature review. BMC Gastroenterol. 2022;22(1):99. 35246051 10.1186/s12876-022-02179-z PMC8895667

[4] Wang WL Farris AB Lauwers GY Deshpande V. Autoimmune pancreatitis-related cholecystitis: A morphologically and immunologically distinctive form of lymphoplasmacytic sclerosing cholecystitis. Histopathology. 2009;54(7):829–36. 19635102 10.1111/j.1365-2559.2009.03315.x

[5] Wallace ZS Katz G Hernandez-Barco YG Baker MC. Current and future advances in practice: IgG4-related disease. Rheumatol Adv Pract. 2024;8(2):rkae020. 38601138 10.1093/rap/rkae020 PMC11003820

